# Prevalence of extended-spectrum beta-lactamase producing bacteria from animal origin: A systematic review and meta-analysis report from India

**DOI:** 10.1371/journal.pone.0221771

**Published:** 2019-09-04

**Authors:** Suresh P. Kuralayanapalya, Sharanagouda S. Patil, Sudhakar Hamsapriya, Rajamani Shinduja, Parimal Roy, Raghavendra G. Amachawadi

**Affiliations:** 1 ICAR-National Institute of Veterinary Epidemiology and Disease Informatics (NIVEDI), Yelahanka, Bengaluru, India; 2 Department of Clinical Sciences, College of Veterinary Medicine, Kansas State University, Manhattan, KS, United States of America; Panstwowy Instytut Weterynaryjny - Panstwowy Instytut Badawczy w Pulawach, POLAND

## Abstract

Antimicrobial resistance (AMR) due to the emergence and spread of extended-spectrum beta-lactamase (ESBL) producing bacteria are becoming a serious global public health concern. This article aims to assess the overall prevalence of ESBLs among animals in India, with year-wise, zone-wise and species-wise stratification. Systematic search from PubMed, Google Scholar and J-Gate Plus was carried out and 24 eligible articles from 2013–2019 in India were retrieved. The R Open source Scripting software was used to perform statistical analysis. The overall prevalence of ESBLs among animals in India was 9%. The pooled prevalence of ESBLs in animals were 26, 11, 6 and 8% for north, east, south and central zones, respectively. The reported prevalence of ESBLs in animals were 12, 5, 8, 8, 12, 13 and 33% were reported for the years 2013, 2014, 2015, 2016, 2017, 2018, 2019 respectively. The species-wise stratified results showed a predominance of ESBL producing *Klebsiella pneumoniae* strains (11%) when compared to *Escherichia coli* and *Pseudomonas* spp. which were 7% and 5%, respectively. The prevalence data generated could be utilized in infection control and in antibiotic use management decisions for developing appropriate intervention strategies.

## Introduction

Antimicrobial resistance (AMR) has been universally recognized as an emerging global problem to public health. Although the prevalence of AMR is sporadic, it is widespread in the Asian region. India, located in the southern part of Asia, marks a high, immeasurable burden of AMR among livestock due to poor documentation, sub-standard regulations with a shortfall in forbidding protocol enforcement [1]. This study aims to estimate the pooled prevalence of Extended-spectrum *β*-lactamases (ESBLs) in India by conducting systematic review and meta-analysis with 23 available research articles under epidemiological study design. Beta-Lactam antimicrobial agents are the most favored class of antimicrobials for the treatment of bacterial infections, hence becoming the main cause of resistance to *β*-lactam antibiotics, globally [2]. Prevalence of ESBLs producing *Klebsiella* is becoming a major concern in China, Korea, Japan and India [3]. ESBLs enzymes are produced by the gram-negative bacteria to incur resistance against the *β*-lactams. *Klebsiella pneumoniae* and *Escherichia coli* are the main gram-negative bacteria producing ESBLs [4]. However *Proteus mirabilis*, *Enterobacter* spp., *Salmonella*, *Acinetobacter baumannii*, and *Pseudomonas aeruginosa* also produce ESBLs to acquire resistance [5]. The incessant liability of gram-negative strains to a myriad *β*-lactams has begotten rapid and vigorous production and mutation of *β*-lactamases in these bacteria, hence, incurring resistance against the newly developed *β*-lactam antibiotics [2]. Treatment for these disease causing multidrug-resistant (MDR) organisms is a therapeutic challenge. The risk factors for developing infection with ESBL-producing organisms include indiscriminate and off-label use of antibiotics [6]. At present, animals without any recognized risk factor for multidrug-resistant organisms are found to have ESBL-producing organisms. Hence, diagnosis of ESBL-producing organisms has become vital [7]. MDRs are posing a treatment challenge, and a major cause of morbidity and mortality worldwide [1]. Unfortunately, India, being a developing country, does not have an adequate surveillance system that could track indiscriminate use or consumption of antibiotics in livestock populations. This meta-analysis will improve our understanding of the distribution of ESBLs in India. A set of similar events for which a study is conducted is called a population, in our study it refers to poultry, bovine and birds. The outcome of our study would indicate the prevalence of ESBLs by zone, year and species in India. It is a quantitative, epidemiological study designed to systematically assess the previous research studies to derive the conclusions of this research [8]. This study highlighted the prevalence of ESBL from the time period 2013–2019, with zone-wise and species-wise prevalence of ESBLs in India. A priori protocol was followed for this study with reference to a work done by Bulabula and co-workers [9]. To our knowledge, this is the first meta-analysis report from India on animals, which would aid in updating the national treatment guidelines for ESBL infections among animals.

## Materials and methods

### Literature search

A Systematic search was conducted in “Pub Med”, “Google Scholar” and “J-Gate-Plus” databases from Jan 2013 to May 2019 using the search terms “ESBL”, “prevalence”, “India”, “Animals”, “Poultry”, “Cattle” and “Bovine” in combinations. Bibliographies of eligible studies were also manually searched to identify additional significant articles. A comprehensive search was conducted to ensure none of the research were missed out. The search was restricted to articles published in English.

### Study selection criteria

All the articles that described the frequency of ESBL producing pathogens among the total isolates from animal samples (clinical/healthy) were considered eligible and included in the study. The qualified articles described the specific laboratory methods used to identify the ESBL producing pathogen along with species of the ESBL producing organism (Table 1). All the enrolled studies were restricted to India. Review articles, case reports and outbreaks were excluded.

**Table 1:** Characteristics of studies included in the review.

Author and year of publication	State	Country	Sample type	Number of ESBL positive samples/Total number of samples (% prevalence)	Methodology	ESBL producing species
**Bandyopadhyay et al. 2018 [25]**	West Bengal	India	Bovine milk samples	12/424	PCR-based detection of major ESBL blaCTX-M-15 gene	ESBL producing K. pneumoniae
**Bhattacharya et al., 2015 [21]**	West Bengal	India	Meat and meat products	2/80 (2.5%)	Combined Disc Diffusion Test	ESBL producing *E*. *coli*
**Bhave et al., 2019 [28]**	Maharashtra	India	Cloacal swabs of broilers	23/146(15.75%)	PCR-based detection of major ESBL genes (blaTEM, blaSHV, blaCTX-M)	ESBL producing E. coli
**Bhoomika et al., 2016 [10]**	Chhattisgarh	India	Chicken meat	2/65 (3.08%)	Multiplex-polymerase chain reaction for detection of *bla*_TEM_, *bla*_SHV_,and blaCTX-M genes	ESBL genes in *E*. *coli*
**Bhoomika et al., 2016 [10]**	Chhattisgarh	India	Chevon meat	1/38 (2.63%)	Multiplex-polymerase chain reaction for detection of *bla*_TEM_, *bla*_SHV_, and blaCTX-M genes	ESBL genes in *E*. *coli*
**Bhoomika et al., 2016 [10]**	Chhattisgarh	India	Raw milk	6/73 (8.22%)	Multiplex-polymerase chain reaction for detection of *bla*_TEM_, *bla*_SHV_, and blaCTX-M genes	ESBL genes in *E*. *coli*
**Brower et al., 2017 [12]**	Punjab	India	Cloacal swabs from birds	305/1556 (19.60%)	Combination disk method and VITEK 2	ESBL producing *E*. *coli*
**Chauhan et al., 2013 [13]**	Himachal Pradesh	India	Raw milk samples from Doon valley	27/100 (27%)	Double disc diffusion method	ESBL producing *K*. *pneumoniae*
**Das et al., 2017 [15]**	West Bengal	India	Milk samples of subclinical mastitis infected cattle	24/50 (48%)	PCR-based detection of major ESBL genes (*bla*_TEM_, *bla*_SHV_, *bla*_CTX-M_)	ESBL producing gram negative isolates
**Dewangan et al., 2017 [16]**	Chhattisgarh	India	Chevon meat	8/126 (6.35%)	Phenotypic detection of ESBL	ESBL producing *E*. *coli*
**Dewangan et al., 2017 [16]**	Chhattisgarh	India	Raw milk samples	8/104 (7.69%)	Phenotypic detection of ESBL	ESBL producing *E*. *coli*
**Kar et al., 2015 [22]**	West Bengal	India	Fecal samples from poultry	16/170 (9.41%)	Combination disc method and ESBL E-test	ESBL producing *E*. *coli*
**Kar et al., 2015 [22]**	West Bengal	India	Milk samples from cattle	2/135 (1.48%)	Combination disc method and ESBL E-test	ESBL producing *E*. *coli*
**Karuppasamy et al., 2015 [23]**	Mizoram	India	Raw milk samples	7/35 (20%)	Kirby-Bauer disc diffusion method	ESBL producing *E*. *coli* and *K*. *pneumoniae*
**Koovapra et al., 2016 [40]**	West Bengal	India	Bovine milk samples	7/159 (4.40%)	Combination disc diffusion test and ESBL Etest	ESBL producing *K*. *pneumoniae*
**Koovapra et al., 2016 [40]**	Jharkhand	India	Bovine milk samples	10/78 (12.82%)	Combination disc diffusion test and ESBL Etest	ESBL producing *K*. *pneumoniae*
**Koovapra et al., 2016 [40]**	Mizoram	India	Bovine milk samples	6/103 (5.82%)	Combination disc diffusion test and ESBL Etest	ESBL producing *K*. *pneumoniae*
**Lalzampuia et al., 2013 [16]**	Mizoram	India	Fecal samples of pigs	7/138 (5.07%)	PCR based detection of ESBLs genes	ESBL genes in *E*. *coli*
**Lalzampuia et al., 2013 [17]**	Mizoram	India	Fecal samples of poultry birds	4/102 (3.92%)	PCR based detection of ESBLs genes	ESBL genes in *E*. *coli*
**Lalzampuia et al., 2014 [18]**	Mizoram	India	Fecal samples of poultry birds	1/11 (9.09%)	PCR based detection of ESBLs genes	ESBL genes in *K*. *pneumoniae*
**Mahanti et al., 2017 [14]**	West Bengal	India	Cloacal swabs from healthy broiler, indigenous, and kuroiler birds	33/307 (10.75%)	PCR-based detection of major ESBL genes (*bla*_TEM_, *bla*_SHV_, *bla*_CTX-M_)	ESBL producing *K*. *pneumoniae*
**Mandakini et al., 2015 [24]**	Mizoram	India	Fecal samples of piglets suffering from diarrhea	43/170 (25.29%)	Double disc synergy test	ESBL producing *E*. *coli*
**Nirumapa et al., 2018 [26]**	Uttar	India	Fecal samples of pigs	243/741(32.79%)	Double disc diffusion method and Hi-comb MIC test strip	ESBL producing *E*. *coli*
**Raj et al., 2019 [29]**	Karnataka	India	Food-animal environment	12/43(27.90%)	PCR-based detection of major ESBL blaCTX-M	ESBL producing E. coli
**Rasheed et al., 2014 [19]**	Telangana	India	Unpasteurized milk of buffalo	2/30 (6.67%)	Phenotypic Confirmatory Disc Diffusion Test	ESBL producing *E*. *coli*
**Rasheed et al., 2014 [19]**	Telangana	India	Raw chicken	0/30 (0%)	Phenotypic Confirmatory Disc Diffusion Test	ESBL producing *E*. *coli*
**Rasheed et al., 2014 [19]**	Telangana	India	Fresh raw meat of sheep	1/30 (3.33%)	Phenotypic Confirmatory Disc Diffusion Test	ESBL producing *E*. *coli*
**Samanta et al., 2015 [31]**	West Bengal	India	Samples from backyard and farmed poultry	23/360 (6.39%)	PCR-based detection of major ESBL genes (*bla*_TEM_, *bla*_SHV_, *bla*_CTX-M_)	ESBL producing *E*. *coli*
**Sharif et al., 2017 [19]**	Andhra Pradesh	India	Rectal swab samples from healthy dogs	2/92 (2.17%)	Combination disc method	ESBL producing *Pseudomonas* species
**Sharif et al., 2017 [20]**	Andhra Pradesh	India	Rectal swab samples from diarrheic dogs	5/44 (11.36%)	Combination disc method	ESBL producing *Pseudomonas* spp.
**Shrivastav et al., 2016 [41]**	Madhya Pradesh	India	Cecal swab samples in healthy broilers	135/400 (33.75%)	Combined disc diffusion test, DDST, Enz MIC strip	ESBL producing *E*. *coli*
**Tewari et al., 2018 [27]**	Assam	India	Fecal samples of livestock	10/48 (20.83%)	PCR-based detection	ESBL producing E. coli
**Tewari et al., 2019 [30]**	Meghalaya and Assam	India	Fecal samples of livestock	24/32 (75%)	PCR-based detection	ESBL producing E. coli

### Data extraction

For consistency, data was extracted independently by two people from selected articles. The data extracted from qualified studies included year of publication, first author, location where study was conducted, total sample size, strains detected ESBL positive, and method used for confirmation of ESBL producing pathogen. Any inconsistency in data collection was rectified by re-checking the articles for accuracy.

### Quality assessment

Since it is a prevalence study, use of Newcastle-Ottawa scale is not recommend. However, quality assessment of the study was done on fixed rating scale. This scale includes evaluation of study selection, comparability and outcome, with each section having maximum number of stars as 5, 3 and 2 respectively. Hence, the overall quality assessment has a maximum score of 10 and minimum score for inclusion is 3 stars. Table 2 shows the risk of bias assessment for the studies included in quantitative synthesis.

**Table 2:** Meta-analysis of ESBL prevalence in animals from India.

Sl no.	Parameters	Period	Number of Articles	Number of Studies	Total Events	Pooled Prevalence(With 95% Confidence Interval)	*I*^2^ Value (%)	*τ*^2^ Value	p Value
**1.**	ESBL prevalence in Animals	2013–2019	17	27	6020	10 (7–15)	94	0.8090	p<0.01
**Year-Wise**									
**1.**	Year 2013	2013	2	2	238	12 (2–62%)	95	1.7709	p<0.01
**2.**	Year 2014	2014	2	5	203	5 (2–9%)	0	0	p = 0.81
**3.**	Year 2015	2015	5	6	950	8 (3–18%)	91	1.1334	p<0.01
**4.**	Year 2016	2016	3	7	916	8 (4–16%)	93	0.6677	p<0.01
**5.**	Year 2017	2017	5	7	2279	12 (6–22%)	91	1.0612	p<0.01
**6.**	Year 2018	2018	3	3	1213	13(3–55%)	95	1.6353	p<0.01
**7.**	Year 2019	2019	3	3	221	33(13–81)	97	0.6148	p<0.01
**Zone-Wise**									
**1.**	North Zone	2013–2017	2	2	2397	26 (19–36%)	96	0.0711	p<0.01
**2.**	East Zone	2013–2017	10	14	1978	11 (6–18%)	95	0.9394	p<0.01
**3.**	West Zone	-	0	0	-	-	-	-	-
**4.**	South Zone	2014–2017	2	5	226	6 (3–11%)	36	0.2815	p = 0.20
**5.**	Central Zone	2016–2017	3	6	806	8 (4–18%)	92	0.6864	p<0.01
**Species-Wise**									
**1.**	*Escherichia coli*	2013–2017	11	17	4526	9 (6–15%)	92	0.8028	p<0.01
**2.**	*K*. *pneumoniae*	2013–2017	4	6	758	10 (6–19%)	85	0.3852	p<0.01
**3.**	*Pseudomonas* spp.	2017	1	2	136	5 (1–24%)	76	1.0345	p = 0.04

### Statistical analysis

Meta-analysis for the prevalence of ESBL producing pathogens among animal samples were conducted using the R Open source scripting software (version 3.4.3, R Foundation for Statistical Computing, Vienna, Austria. https://www.R-project.org/) [10]. The inbuilt packages used for analysis were Metafor and Meta R packages.

In the analysis, both random effect and fixed effect model were used to calculate the pooled prevalence of ESBL and *I*^2^ statistic (to measure inconsistency). The *τ*^2^ statistic was also calculated to measure the heterogeneity. Further, sub-group analysis was performed to reduce heterogeneity. In the present study, the data was stratified based on: year-wise (2013–2019) zone-wise (North, East, West, South and Central zones) and species-wise (*E*. *coli*, *Pseudomonas* spp., and *K*. *pneumoniae*).

## Results

### Distribution and characteristics of articles describing ESBLs in India

**Fig 1:**
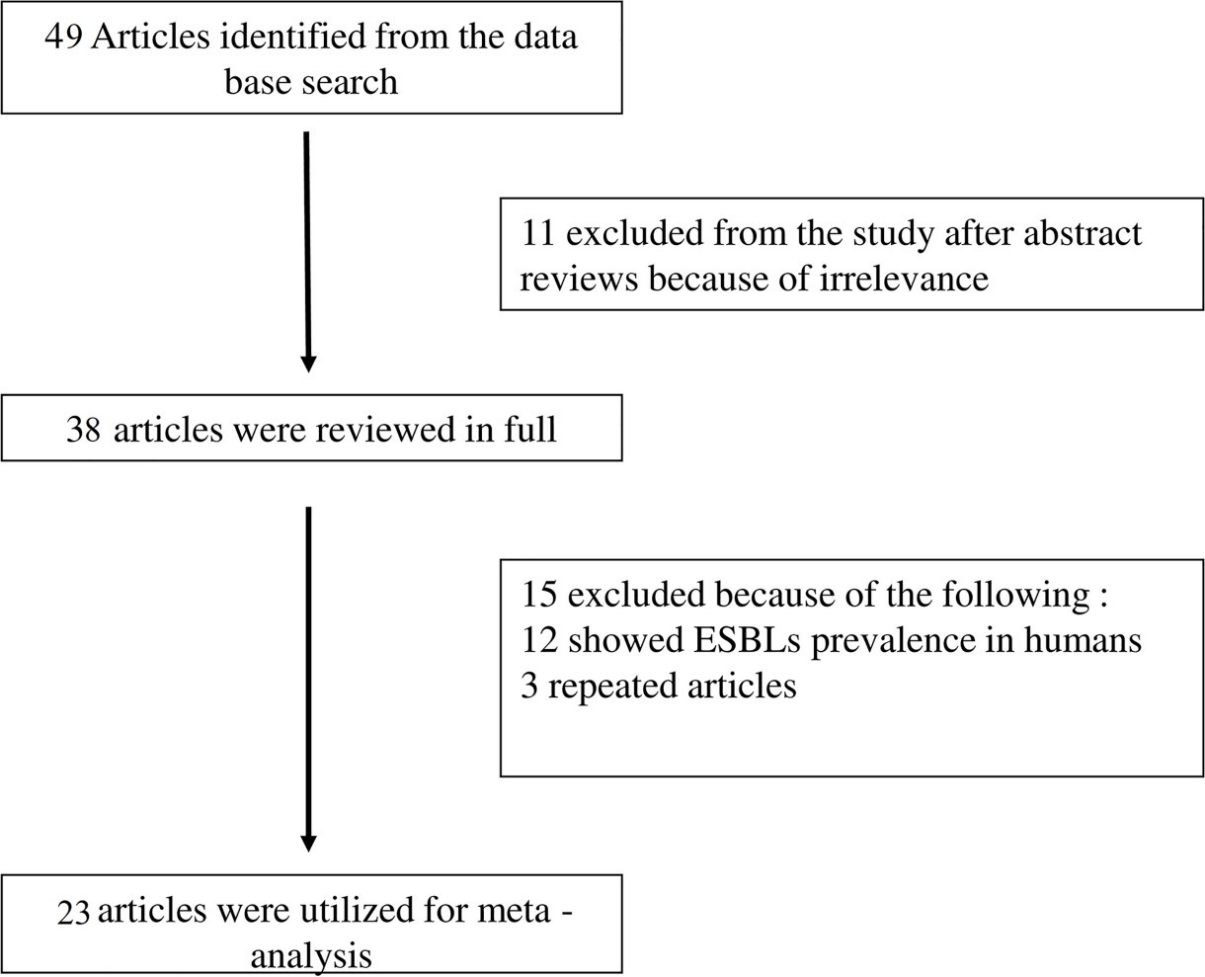
The flow diagram of study selection process.

The electronic database searches returned 32 potential articles based on the keyword search. Review articles studying the ESBL prevalence in humans were excluded. A total of 23 articles were selected suitable for the study. The flowchart of systematic article selection is shown in Fig 1. All the articles included in the study described the prevalence of ESBL producing pathogens isolated from animals/animal samples from India. The maximum number of studies on this subject were found in the eastern zone followed by central, south and north zone. No studies were found from western zone of India. In total, 20 studies were on ESBLs produced by *Escherichia coli*, 6 on ESBLs produced by *K*. *pneumoniae* and 2 on ESBLs produced from *Pseudomonas* spp. The animal samples studied in the articles mainly included meat samples, milk samples, rectal swabs, cecal swabs and cloacal swabs from poultry birds, sheep, pig and cattle.

### Pooled prevalence of ESBLs in animal samples

**Fig 2:**
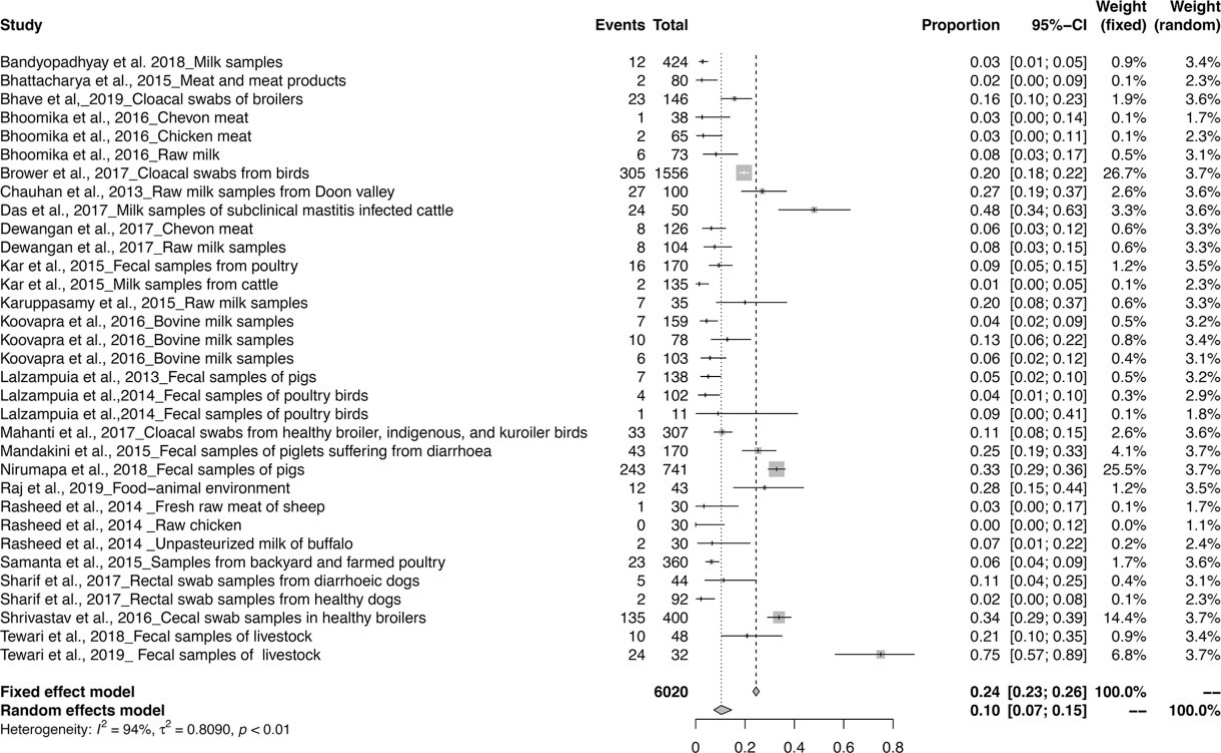
Forest plot of ESBL prevalence in India from 2013–2019.

**Fig 3:**
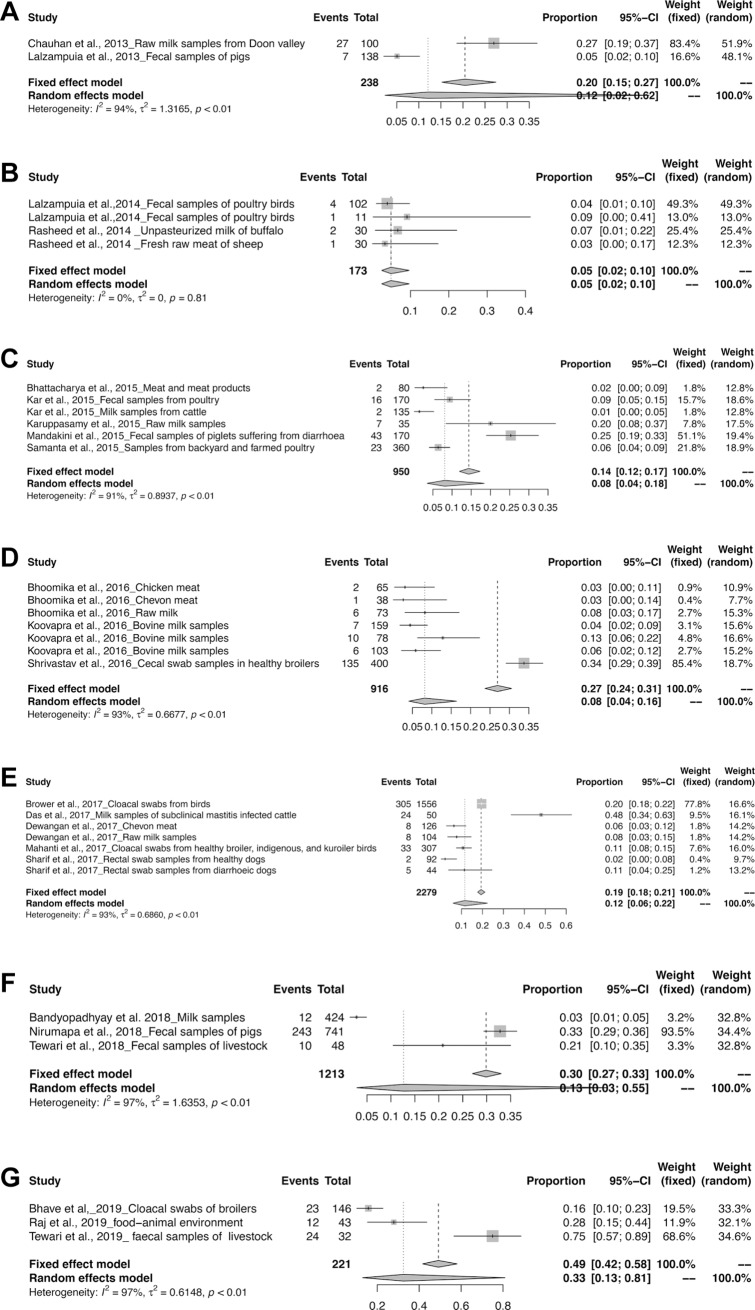
Forest plots of ESBL prevalence in (a) 2013; (b) 2014; (c) 2015; (d) 2016; (e) 2017; (f) 2018; and (g) 2019.

**Fig 4:**
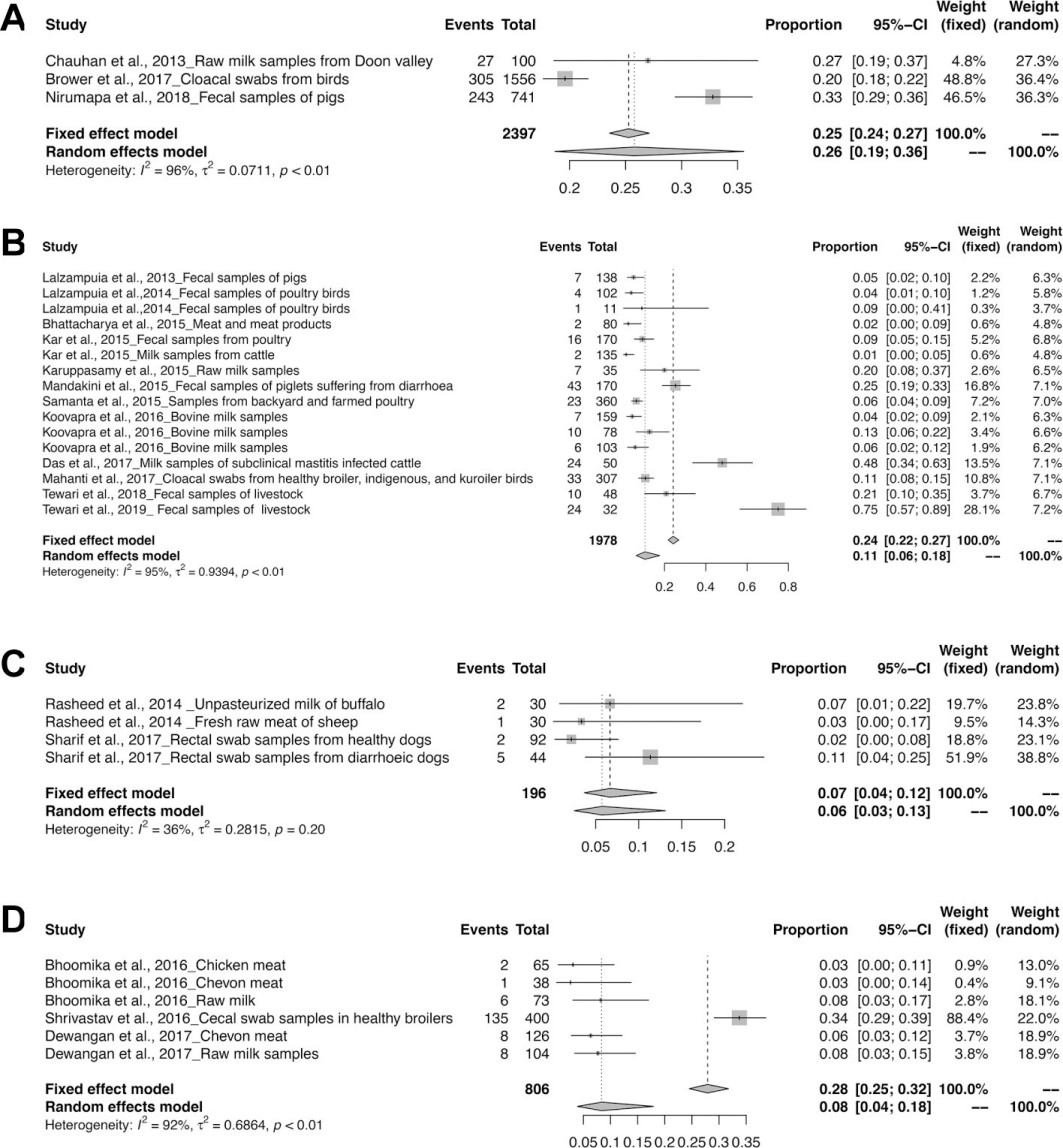
Forest plots of ESBL prevalence in (a) north-zone; (b) east-zone; (c) south-zone; and (d) central-zone.

**Fig 5:**
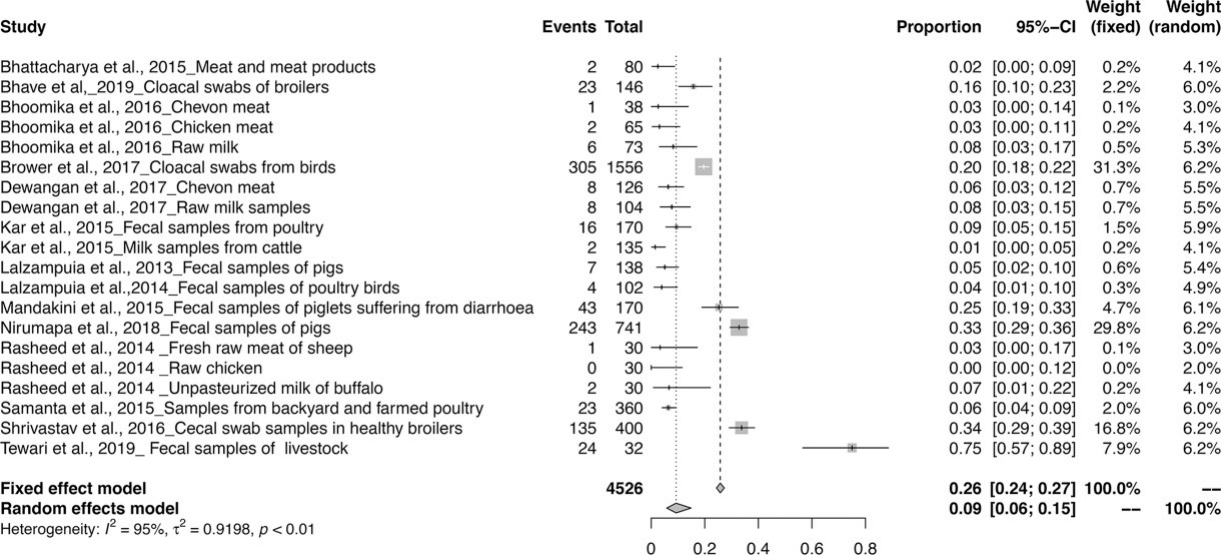
Forest plot of *E. coli* producing ESBL prevalence.

**Fig 6:**
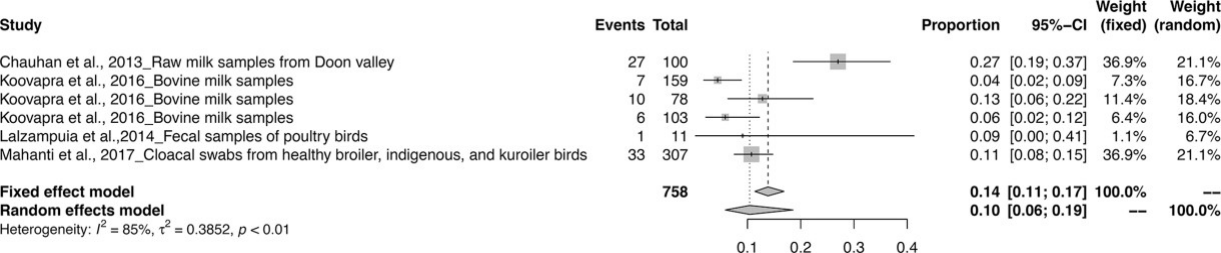
Forest plot of *K. pneumonia* producing ESBL prevalence.

**Fig 7:**
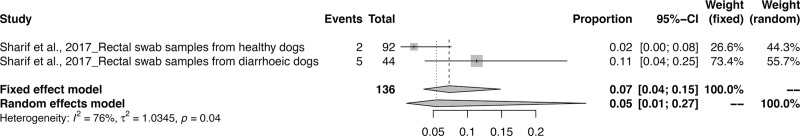
Forest plot of *Pseudomonas spp*. producing ESBL prevalence.

The meta-analysis revealed the overall pooled prevalence of ESBL in animals to be 9% (95% CI: 6–13%; *τ*^2^ = 0.6654; *P* < 0.01**). The prevalence estimates of ESBL producing pathogens in India is depicted in the forest plot in Fig 2, which also displays the author, year, samples, events and total samples [11–20]. In order to reduce the heterogeneity, the studies on ESBL producing isolates were categorized by Year, Zone and Species-wise (Table 3). The pooled prevalence of ESBL producing pathogens in animals were 12, 5, 8, 8, 12, 13 and 33% for the years 2013, 2014, 2015, 2016, 2017, 2018 and 2019 respectively, as depicted in the forest plot [20–30] in (Fig 3A–3G). The zone-wise prevalence percentage of ESBLs were 26, 11, 6 and 8% for the north, east, south and central zones are shown in (Fig 4A–4D). The species-wise prevalence of ESBLs were found to be 9, 10 and 5% for *E*.*coli*, *K*. *pneumoniae* and *Pseudomonas spp*. respectively. Figs 5–7 explains the forest plot of species-wise Meta-analysis.

**Table 3:** Risk of bias assessment for studies included in the quantitative synthesis.

Author and year of publication	Selection		Comparability	Outcome	Overall Quality Assessment score
	Representativeness of the sample	Ascertainment of exposure		Assessment of outcome	
**Bandyopadhyay et al. 2018 [25]**	*Truly representative bovine milk samples	**ESBL production confirmed by PCR	Study did not control for other factors	*Independent blind assessment	3
**Bhattacharya et al., 2015 [20]**	*Truly representative Meat and meat products with antibiotic resistance	*ESBL production diagnosed by Combined Disc Diffusion Test	Study did not control for other factors	*Independent blind assessment	3
**Bhave et al., 2019 [28]**	*Truly representative Cloacal swabs from broiler	*ESBL production diagnosed by Combined Disc Diffusion Test	Study did not control for other factors	*Independent blind assessment	4
**Bhoomika et al., 2016 [10]**	*Truly representative of chicken meat samples with antibiotic resistance	**Chicken meat samples diagnosed with clinical isolates producing ESBL confirmed by Multiplex PCR	Study did not control for other factors	*Independent blind assessment	4
**Bhoomika et al., 2016 [10]**	*Truly representative of chevon meat samples with antibiotic resistance	**Chevon meat samples diagnosed with clinical isolates producing ESBL confirmed by Multiplex PCR	Study did not control for other factors	*Independent blind assessment	4
**Bhoomika et al., 2016 [10]**	*Truly representative of raw milk samples with antibiotic resistance	**Raw milk samples diagnosed with clinical isolates producing ESBL confirmed by Multiplex PCR	Study did not control for other factors	*Independent blind assessment	4
**Brower et al., 2017 [11]**	*Truly representative Cloacal swabs from birds with antibiotic resistance	*ESBL production diagnosed by Combination disk method and VITEK 2	Study did not control for other factors	*Independent blind assessment	3
**Chauhan et al., 2013 [12]**	*Truly representative Raw milk samples from Doon valley with antibiotic resistance	*ESBL production diagnosed by Double disc diffusion method	Study did not control for other factors	*Independent blind assessment	3
**Das et al., 2017 [14]**	*Truly representative of sub-clinical mastic milk samples with antibiotic resistance	**Sub-clinical mastic milk samples diagnosed with clinical isolates producing ESBL confirmed by PCR	Study did not control for other factors	*Independent blind assessment	4
**Dewangan et al., 2017 [15]**	*Truly representative Chevon meat with antibiotic resistance	*ESBL production diagnosed by Phenotypic detection of ESBL	Study did not control for other factors	*Independent blind assessment	3
**Dewangan et al., 2017 [15]**	*Truly representative Raw milk samples with antibiotic resistance	*ESBL production diagnosed by Phenotypic detection of ESBL	Study did not control for other factors	*Independent blind assessment	3
**Kar et al., 2015 [21]**	*Truly representative Fecal samples from poultry with antibiotic resistance	*ESBL production diagnosed by Combination disc method and ESBL E-test	Study did not control for other factors	*Independent blind assessment	3
**Kar et al., 2015 [21]**	*Truly representative Milk samples from cattle with antibiotic resistance	*ESBL production diagnosed by Combination disc method and ESBL E-test	Study did not control for other factors	*Independent blind assessment	3
**Karuppasamy et al., 2015 [22]**	*Truly representative Raw milk samples with antibiotic resistance	*ESBL production diagnosed by Kirby Bauer disc diffusion test	Study did not control for other factors	*Independent blind assessment	3
**Koovapra et al.,2016 [33]**	*Truly representative Bovine milk samples with antibiotic resistance	ESBL production diagnosed by Combination disc diffusion test and ESBL Etest	Study did not control for other factors	*Independent blind assessment	3
**Lalzampuia et al., 2013 [16]**	*Truly representative Fecal samples of pigs with antibiotic resistance	**Pigs with history of diarrhea diagnosed with clinical isolates producing ESBL confirmed by PCR	Study did not control for other factors	*Independent blind assessment	4
**Lalzampuia et al., 2014 [17]**	*Truly representative Fecal samples of poultry birds with antibiotic resistance	**Poultry birds with history of diarrhea diagnosed with clinical isolates producing ESBL confirmed by PCR	Study did not control for other factors	*Independent blind assessment	4
**Lalzampuia et al., 2014 [17]**	*Truly representative Fecal samples of poultry birds with antibiotic resistance	**Poultry birds with history of diarrhea diagnosed with clinical isolates producing ESBL confirmed by PCR	Study did not control for other factors	*Independent blind assessment	4
**Mahanti et al., 2017 [13]**	*Truly representative Cloacal swabs from healthy broiler, indigenous, and kuroiler birds with antibiotic resistance	**ESBL production confirmed by PCR	Study did not control for other factors	*Independent blind assessment	4
**Mandakini et al., 2015 [23]**	*Truly representative Fecal samples of piglets suffering from diarrhea with antibiotic resistance	*ESBL production diagnosed by Double disc synergy test	Study did not control for other factors	*Independent blind assessment	3
**Nirumapa et al., 2018 [26]**	*Truly representative Fecal samples of pigs	** ESBL production diagnosed by Double disc diffusion method and Hi-comb MIC test strip	Study did not control for other factors	*Independent blind assessment	3
**Raj et al., 2019 [29]**	* Truly representative Food-animal environment	**ESBL production diagnosed by PCR	Study did not control for other factors	*Independent blind assessment	3
**Rasheed et al., 2014 [18]**	*Truly representative Unpasteurized milk of buffalo with antibiotic resistance	*ESBL production diagnosed by Phenotypic Confirmatory Disc Diffusion Test	Study did not control for other factors	*Independent blind assessment	3
**Rasheed et al., 2014 [18]**	*Truly representative Raw chicken with antibiotic resistance	*ESBL production diagnosed by Phenotypic Confirmatory Disc Diffusion Test	Study did not control for other factors	*Independent blind assessment	3
**Rasheed et al., 2014 [18]**	*Truly representative Fresh raw meat of sheep with antibiotic resistance	*ESBL production diagnosed by Phenotypic Confirmatory Disc Diffusion Test	Study did not control for other factors	*Independent blind assessment	3
**Samanta et al., 2015 [24]**	*Truly representative Samples from backyard and farmed poultry with antibiotic resistance	**ESBL production confirmed by PCR	Study did not control for other factors	*Independent blind assessment	4
**Sharif et al., 2017 [19]**	*Truly representative Rectal swab samples from healthy dogs with antibiotic resistance	*ESBL production diagnosed by Combined Disc Diffusion Test	Study did not control for other factors	*Independent blind assessment	3
**Sharif et al., 2017 [19]**	*Truly representative Rectal swab samples from diarrheic dogs with antibiotic resistance	*ESBL production diagnosed by Combined Disc Diffusion Test	Study did not control for other factors	*Independent blind assessment	3
**Shrivastav et al., 2016 [35]**	*Truly representative Cecal swab samples in healthy broilers with antibiotic resistance	*ESBL production diagnosed by CDDT, DDST and Enz MIC strip in Healthy broilers	Study did not control for other factors	*Independent blind assessment	3
**Tewari et al., 2018 [27]**	*Truly representative Fecal samples of livestock	**ESBL production confirmed by PCR	Study did not control for other factors	*Independent blind assessment	3
**Tewari et al., 2019 [30]**	*Truly representative Fecal samples of livestock	**ESBL production confirmed by PCR	Study did not control for other factors	*Independent blind assessment	3

PCR, Polymerase chain Reaction; CDDT, Combined disc diffusion test; DDST, Double disc synergy test, Enz MIC strip, Enz Minimum Inhibitory Concentration strip; E test, Epsilometer test.

(*)Stars represent the number of points awarded for the category;

* = 1,

** = 2.

## Discussion

Our study revealed that, the ESBL producing clinical isolates in India may not be very high, nonetheless it is significant. These drug-resistant pathogens are a serious concern worldwide as they are associated with increase in morbidity and mortality rate due to infections they cause [31]. Extended-Spectrum Beta-Lactamases are produced by species of bacteria in order to inactivate antibiotics, causing antibiotic resistance. Beta-lactamase seems to be the prime cause in multidrug resistant (MDR) *E*. *coli* strains. Early detection of *E*. *coli* that produce beta lactamase is necessary in order to prevent MDR *E*. *coli* from spreading [32]. Activity of ESBLs caused by different beta-lactamases resulted in resistant genes within the farm [33]. The strains that were isolated showed that a small portion of the resistant genes were present in one farm [4]_._ The steep rise in income and the growing population has driven an increase in demand for animal products in India. India is one of the top consumers of antibiotics worldwide, it accounts for about 3% of global consumption which is estimated to double by 2030. This could be due to the non-therapeutic use of antibiotics in cases of prophylaxis and growth promotion [34]. Currently, the usage of antibiotics is high in poultry, swine and cattle production as compared to that being used by the human population [35–36].

To address the concern of antimicrobial resistant bacteria, it is crucial to raise awareness of the problem by collecting data on antibiotic resistance from various countries and regions. The paucity of studies available from India affirms attention for future research. To our knowledge, this is the first meta-analysis regarding the magnitude of the ESBL problem in Indian animal population. From the 23 articles chosen in the study, the overall pooled prevalence of ESBL producing isolates from the animal samples was found to be 10%. In Asia, high rates of ESBL producing *Enterobacteriaceae* are seen with variation in the prevalence and the genotype of the ESBL producing isolates over the large geographical area [30].

The prevalence of ESBL producing isolates were 12, 5, 8, 8, 12, 13 and 33% for the years 2013, 2014, 2015, 2016, 2017, 2018 and 2019 respectively, indicating an increase in the percent drug resistance since 2014 to 2019. The pooled prevalence of ESBL producing isolates was determined zone-wise and North zone showed a higher prevalence rate in comparison to other zones. Nonetheless, no studies on prevalence of ESBL producing isolates for animal samples from the Western zone of India are reported. Prevalence of species-wise classification was found to be 9, 10 and 5% for *E*. *coli*, *K*. *pneumoniae* and *Pseudomonas* spp. respectively, signifying that the ESBL producing *K*. *pneumoniae* is the most predominant ESBL producing isolate in India.

A study conducted in the intensive care units (ICUs) of an Indian hospital concluded that there is a need for constant surveillance to detect resistant bacterial strains, strict guidelines on antibiotic therapy, and effective infection control measures in order to reduce the spread of antibiotic resistant bacteria. The same study also revealed that there is a high number of ESBL producing *E*. *coli* in the ICUs of that hospital [31]. A study with pediatric and neonatal patients estimated the number of poor outcomes and indicated the association of blood stream infections (BSIs) with Extended-Spectrum Beta-Lactamase- producing *Enterobacteriaceae* (ESBL-PE). The results showed a high prevalence of BSIs due to ESBL-PE and increase in neonatal mortality [37–39]. A study from Germany demonstrated that direct transfer of ESBL-producing E. *coli* could occur between livestock and the farm workers who were in close contact with farm animals. The study also suggests an existence of epidemiological links between livestock and farm workers. A high prevalence of ESBL-producing *E*. *coli* in pig and cattle farms emphasizes the fact that livestock animals are a constant source for these potential human pathogens [33, 40–41].

Our research findings does have some minor limitations, which includes the lack of sufficient information on the prevalence of ESBL producers from different animal species. Upon advanced literature survey, we could find only a few articles that addressed the prevalence of ESBLs in animals.

## Conclusion

India being a developing country, has the highest burden of bacterial infections. Hence, to combat this downfall, antibiotics are used widely and indiscriminately. The overuse, lack of awareness and non-therapeutic use of antibiotics is driving an increase in the antibiotic resistance among animals. This meta-analysis, indicated that the pooled prevalence of ESBLs for animals in India is not high, however, the overall prevalence remains significant at 10%. Additionally, only little information is currently available that addresses the prevalence of ESBLs in animals in India. The paucity of data on the clinical outcomes, magnitude and prevalence of the resistant ESBLs, calls for active surveillance which can help understand the epidemiology of ESBL burden in India. Furthermore, emphasis on awareness programs, personal and environmental hygiene should be implemented to stop and manage the spread of ESBLs to the animals and environment. Further studies are needed to better understand the complexity of the AMR problem in animal and human population.

## Supporting information

S1 File PRISMA checklist. (DOC)

S2 File Listed references for underlying data. (DOCX)

## References

[1] WHO-Ministry of health and family welfare: Antimicrobial resistance and its containment in India. World Health Organization; 2016. [Online at: http://www.searo.who.int/india/topics/antimicrobial_resistance/amr_containment.pdf?ua=1]

[2] Sarojamma V, Ramakrishna V. Prevalence of ESBL-producing *Klebsiella pneumoniae* isolates in tertiary care hospital. ISRN Microbiol. 2011;2011:318348 10.5402/2011/318348 23724303 PMC3658478

[3] Hawkey PM. Prevalence and clonality of extended‐spectrum β‐lactamases in Asia. Clin Microbiol Infect. 2008;14:159–65. 10.1111/j.1469-0691.2007.01855.x 18154540

[4] Ibrahim DR, Dodd CE, Stekel DJ, Ramsden SJ, Hobman JL. Multidrug resistant, extended spectrum β-lactamase (ESBL)-producing *Escherichia coli* isolated from a dairy farm. FEMS Microbiol Ecol. 2016;92:fiw013 10.1093/femsec/fiw013 26850161

[5] Segar L, Kumar S, Joseph NM, Sivaraman U. Prevalence of extended spectrum beta-lactamases among *Enterobacteriaceae* and their anti-biogram pattern from various clinical samples. Asian J Pharma Clin. Res 2015;8(5):237–40.

[6] Nóbrega DB, Brocchi M. An overview of extended-spectrum beta-lactamases in veterinary medicine and their public health consequences. J Infect Dev Ctries. 2014;8:954–60. 10.3855/jidc.4704 25116659

[7] Carattoli A. Animal reservoirs for extended spectrum β‐lactamase producers. Clin Microbiol Infect. 2008; 14:117–23. 10.1111/j.1469-0691.2007.01851.x 18154535

[8] Haidich AB. Meta-analysis in medical research. Hippokratia. 2010;14:29–37. 21487488 PMC3049418

[9] Bulabula ANH, Dramowski A, Mehtar S. Maternal colonization or infection with extended-spectrum beta-lactamase-producing *Enterobacteriaceae* in Africa: A systematic review and meta-analysis. Int J Infect Dis. 2017;64:58–66. 10.1016/j.ijid.2017.08.015 28890179

[10] R Open source scripting software (version 3.4.3, R Foundation for Statistical Computing, Vienna, Austria. https://www.R-project.org/).

[11] Bhoomika SS, Patyal A, Gade NE. Occurrence and characteristics of extended-spectrum β-lactamases producing *Escherichia coli* in foods of animal origin and human clinical samples in Chhattisgarh, India. Vet World. 2016;9:996–1000. 10.14202/vetworld.2016.996-1000 27733802 PMC5057040

[12] Brower CH, Mandal S, Hayer S, Sran M, Zehra A, Patel SJ, Kaur R, Chatterjee L, Mishra S, Das BR, Singh P. The prevalence of extended-spectrum beta-lactamase-producing multidrug-resistant *Escherichia coli* in poultry chickens and variation according to farming practices in Punjab, India. Environ Health Perspect. 2017 7;125 10.1289/EHP292 28749780 PMC5744676

[13] Chauhan S, Farooq U, Singh V, Kumar AJ. Determination of prevalence and antibacterial activity of ESBL (Extended Spectrum Beta-lactamases) producing *Klebsiella* species isolated from raw milk of Doon Valley in India. Int Pharma Bio Sci. 2013;4(1):417–23.

[14] Mahanti A, Ghosh P, Samanta I, Joardar SN, Bandyopadhyay S, Bhattacharyya D, Banerjee J, Batabyal S, Sar TK, Dutta TK. Prevalence of CTX-M-Producing *Klebsiella* spp. in Broiler, Kuroiler, and Indigenous Poultry in West Bengal State, India. Microb Drug Res. 2018 4 1;24(3):299–306. 10.1089/mdr.2016.0096 28829687

[15] Das A, Guha C, Biswas U, Jana PS, Chatterjee A, Samanta I. Detection of emerging antibiotic resistance in bacteria isolated from subclinical mastitis in cattle in West Bengal. Vet World. 2017 5;10(5):517 10.14202/vetworld.2017.517-520 28620255 PMC5465765

[16] Dewangan P, Shakya S, Patyal A, Gade NE. Prevalence and molecular characterization of extended-spectrum b-Lactamases (blaTEM) producing Escherichia coli isolated from humans andfoods of animal origin in Chhattisgarh, India. Indian J Ani Res. 2016 7 25;51(2):310–5. 10.18805/ijar.11165

[17] Lalzampuia H, Dutta TK, Warjri I, Chandra R. PCR-based detection of extended-spectrum β-lactamases (bla CTX-M-1 and bla TEM) in *Escherichia coli*, *Salmonella* spp. and *Klebsiella pneumoniae* isolated from pigs in North Eastern India (Mizoram). Indian J Microbiol. 2013 9 1;53(3):291–6. 10.1007/s12088-013-0378-z 24426125 PMC3689405

[18] Lalzampuia H, Dutta TK, Warjri I, Chandra R. Detection of extended-spectrum β-lactamases (blaCTX-M-1 and blaTEM. Vet World. 2014 11 1;7 10.14202/vetworld.2014.1026-31 PMC4774719 27047141

[19] Rasheed MU, Thajuddin N, Ahamed P, Teklemariam Z, Jamil K. Antimicrobial drug resistance in strains of *Escherichia coli* isolated from food sources. Revista do Instituto de Medicina Tropical de São Paulo. 2014 8;56(4):341–6. 10.1590/S0036-46652014000400012 PMC4131821 25076436

[20] Sharif NM, Sreedevi B, Chaitanya RK, Srilatha C. Detection of extended spectrum beta-lactam (ESBL) resistance in *Pseudomonas* species of canine origin. Pharma Innov. 2017 9 1;6:89.

[21] Bhattacharya C, Nibedita D, Pal D. Detection of extended spectrum beta lactamase (ESBL) producing bacteria from meat and meat products in Kolkata, India. IOSR JDMS. 2015;14:52–5. 10.9790/0853-14885255

[22] Kar D, Bandyopadhyay S, Bhattacharyya D, Samanta I, Mahanti A, Nanda PK, Bandyopadhyay S. Molecular and phylogenetic characterization of multidrug resistant extended spectrum beta-lactamase producing *Escherichia coli* isolated from poultry and cattle in Odisha, India. Infect Genet Evol. 2015;29: 82–90. 10.1016/j.meegid.2014.11.003 25445661

[23] Karuppasamy C, Ralte L, Malsawtluangi L, Chawang S. Prevalence of extended spectrum beta lactamase (esbl) producing pathogens in raw milk samples collected from Aizawl town, Mizoram. 2014 10.4172/2168-9547.1000201

[24] Mandakini R, Dutta TK, Chingtham S, Roychoudhury P, Samanta I, Joardar SN, Pachauau AR, Chandra R. ESBL-producing Shiga-toxigenic *E. coli* (STEC) associated with piglet diarrhoea in India. Trop Anim Health Prod. 2015 2 1;47(2):377–81. 10.1007/s11250-014-0731-1 25471364

[25] Bandyopadhyay S, Banerjee J, Bhattacharyya D, Samanta I, Mahanti A, Dutta TK, Ghosh S, Nanda PK, Dandapat P, Bandyopadhyay S. Genomic identity of fluoroquinolone-resistant bla CTX-M-15-Type ESBL and pMAmpC β-lactamase producing *Klebsiella pneumoniae* from buffalo milk, India. Microbial Drug Res. 2018 11 1;24(9):1345–53. 10.1089/mdr.2017.0368 29565231

[26] Nirupama KR, OR VK, Pruthvishree BS, Sinha DK, Murugan MS, Krishnaswamy N, Singh BR. Molecular characterisation of blaOXA-48 carbapenemase-, extended-spectrum β-lactamase-and Shiga toxin-producing *Escherichia coli* isolated from farm piglets in India. J Global Antimicrob Res. 2018 6 1;13:201–5. 10.1016/j.jgar.2018.01.007 29408382

[27] Tewari R, Mitra SD, Venugopal N, Das S, Ganaie F, Sen A, Shome R, Rahman H, Shome BR. Phenotypic and molecular characterization of extended spectrum β-lactamase, ampc β-lactamase and metallo β-lactamase producing *Klebsiella* spp. from farm animals in India. Indian J Anim Res. 2018 10.18805/ijar.B-3599

[28] Bhave S, Kolhe R, Mahadevaswamy R, Bhong C, Jadhav S, Nalband S, Gandhale D, Muglikar D. Phylogrouping and antimicrobial resistance analysis of extraintestinal pathogenic *Escherichia coli* isolated from poultry species. Turkish J Vet Anim Sci. 2019 2 12;43(1):117–26. 10.3906/vet-1808-47

[29] Raj JR, Vittal R, Shivakumaraswamy SK, Deekshit VK, Chakraborty A, Karunasagar I. Presence & mobility of antimicrobial resistance in Gram-negative bacteria from environmental samples in coastal Karnataka, India. Indian J Med Res. 2019 2 1;149(2):290 10.4103/ijmr.IJMR_2088_17 31219097 PMC6563727

[30] Tewari R, Mitra S, Ganaie F, Das S, Chakraborty A, Venugopal N, Shome R, Rahman H, Shome BR. Dissemination and characterization of extended spectrum β-lactamase, AmpC β-lactamase and metallo β-lactamase producing *Escherichia coli* from livestock and poultry in Northeastern India: A molecular surveillance approach. J Global Antimicrob Res. 2019 1 8; 17 (2019) 209–215. 10.1016/j.jgar.2018.12.025 30634056

[31] Samanta I, Joardar SN, Das PK, Sar TK. Comparative possession of Shiga toxin, intimin, enterohaemolysin and major extended spectrum beta lactamase (ESBL) genes in *Escherichia coli* isolated from backyard and farmed poultry. Iran J Vet Res. 2015;16(1):90 27175158 PMC4789247

[32] Boucher HW, Talbot GH, Bradley JS, Edwards JE, Gilbert D, Rice LB, Scheld M, Spellberg B, Bartlett J. Bad bugs, no drugs: No ESKAPE! An update from the Infectious Diseases Society of America. Clin Infect Dis. 2009 1 1;48(1):1–2. 10.1086/595011 19035777

[33] Tewari R, Mitra SD, Ganaie F, Venugopal N, Das S, Shome R, Rahman H, Shome BR. Prevalence of extended spectrum β-lactamase, AmpC β-lactamase and metallo β-lactamase mediated resistance in *Escherichia coli* from diagnostic and tertiary healthcare centers in south Bengaluru, India. 2018 10.18203/2320-6012.ijrms2018128

[34] Dahms C, Hübner NO, Kossow A, Mellmann A, Dittmann K, Kramer A. Occurrence of ESBL-producing *Escherichia coli* in livestock and farm workers in Mecklenburg-Western Pomerania, Germany. PLoS One. 2015 11 25;10(11):e0143326 10.1371/journal.pone.0143326 26606146 PMC4659621

[35] Center for Disease Dynamics, Economics & Policy (CDDEP). 2016. “Antibiotic Use and Resistance in Food Animals.” Washington, D.C, CDDEP. [Online at, https://www.cddep.org/publications/antibiotic_use_and_resistance_food_animals_current_policy_and_recommendations/ ]

[36] Center for Disease Dynamics, Economics & Policy (CDDEP). 2015. “The State Of The World’s Antibiotics 2015” Washington, D.C, CDDEP. [Online at, https://www.cddep.org/wp-content/uploads/2017/06/swa_executive_summary_edits_2016.pdf ]

[37] Dhillon RH, Clark J. ESBLs: A clear and present danger? Crit Care Res Pract. 2012;2012. 10.1155/2012/625170 PMC3135063 21766013

[38] Singh N, Pattnaik D, Neogi DK, Jena J, Mallick B. Prevalence of ESBL in Escherichia coli isolates among ICU patients in a tertiary care hospital. JCDR. 2016 9;10(9):DC19 10.7860/JCDR/2016/21260.8544 27790433 PMC5071933

[39] Flokas ME, Karanika S, Alevizakos M, Mylonakis E. Prevalence of ESBL-producing *Enterobacteriaceae* in pediatric bloodstream infections: A systematic review and meta-analysis. PloS one. 2017 1 31;12(1):e0171216 10.1371/journal.pone.0171216 28141845 PMC5283749

[40] Koovapra S, Bandyopadhyay S, Das G, Bhattacharyya D, Banerjee J, Mahanti A, Samanta I, Nanda PK, Kumar A, Mukherjee R, Dimri U. Molecular signature of extended spectrum β-lactamase producing *Klebsiella pneumoniae* isolated from bovine milk in eastern and north-eastern India. Infect Genet Evol. 2016 10 1;44:395–402. 10.1016/j.meegid.2016.07.032 27473782

[41] Shrivastav A, Sharma RK, Sahni YP, Shrivastav N, Gautam V, Jain S. Study of antimicrobial resistance due to extended spectrum beta-lactamase-producing Escherichia coli in healthy broilers of Jabalpur. Vet World. 201 Nov;9(11):1259 10.14202/vetworld.2016.1259-1263 27956778 PMC5146307

