# Supplementary material for: Prevalence of extended-spectrum beta-lactamase producing bacteria from animal origin: A systematic review and meta-analysis report from India
Source: PLoS One. 2019 Sep 4;14(9):e0221771. doi: 10.1371/journal.pone.0221771 (PMC6726241; doi:10.1371/journal.pone.0221771)
Supplement: S2 File — (DOCX) [file pone.0221771.s002.docx]

1. Bandyopadhyay S, Banerjee J, Bhattacharyya D, Samanta I, Mahanti A, Dutta TK, Ghosh S, Nanda PK, Dandapat P, Bandyopadhyay S. Genomic identity of fluoroquinolone-resistant bla CTX-M-15-Type ESBL and pMAmpC β-lactamase producing *Klebsiella pneumoniae* from buffalo milk, India. Microbial Drug Res. 2018 Nov 1;24(9):1345-53.
2. Bhattacharya C, Nibedita D, Pal D. Detection of extended spectrum beta lactamase (ESBL) producing bacteria from meat and meat products in Kolkata, India. IOSR JDMS. 2015;14:52-5. doi: 10.9790/0853-14885255
3. Bhave S, Kolhe R, Mahadevaswamy R, Bhong C, Jadhav S, Nalband S, Gandhale D, Muglikar D. Phylogrouping and antimicrobial resistance analysis of extraintestinal pathogenic *Escherichia coli* isolated from poultry species. Turkish J Vet Anim Sci. 2019 Feb 12;43(1):117-26. doi: doi:10.3906/vet-1808-47
4. Bhoomika SS, Patyal A, Gade NE. Occurrence and characteristics of extended-spectrum β-lactamases producing *Escherichia coli* in foods of animal origin and human clinical samples in Chhattisgarh, India. Vet World*.* 2016;9:996–1000. doi: [10.14202/vetworld.2016.996-1000](https://doi.org/10.14202/vetworld.2016.996-1000)
5. Brower CH, Mandal S, Hayer S, Sran M, Zehra A, Patel SJ, Kaur R, Chatterjee L, Mishra S, Das BR, Singh P. The prevalence of extended-spectrum beta-lactamase-producing multidrug-resistant *Escherichia coli* in poultry chickens and variation according to farming practices in Punjab, India. Environ Health Perspect. 2017 Jul;125. doi: [10.1289/EHP292](https://doi.org/10.1289/EHP292)
6. Chauhan S, Farooq U, Singh V, Kumar AJ. Determination of prevalence and antibacterial activity of ESBL (Extended Spectrum Beta-lactamases) producing *Klebsiella* species isolated from raw milk of Doon Valley in India. Int Pharma Bio Sci. 2013;4(1):417-23.
7. Das A, Guha C, Biswas U, Jana PS, Chatterjee A, Samanta I. Detection of emerging antibiotic resistance in bacteria isolated from subclinical mastitis in cattle in West Bengal. Vet World. 2017 May;10(5):517. doi: [10.14202/vetworld.2017.517-520](https://doi.org/10.14202/vetworld.2017.517-520)
8. Dewangan P, Shakya S, Patyal A, Gade NE. Prevalence and molecular characterization of extended-spectrum b-Lactamases (blaTEM) producing *Escherichia coli* isolated from humans andfoods of animal origin in Chhattisgarh, India. Indian J Ani Res. 2016 Jul 25;51(2):310-5. doi: [10.18805/ijar.11165](http://dx.doi.org/10.18805/ijar.11165)
9. Kar D, Bandyopadhyay S, Bhattacharyya D, Samanta I, Mahanti A, Nanda PK, Bandyopadhyay S. Molecular and phylogenetic characterization of multidrug resistant extended spectrum beta-lactamase producing *Escherichia coli* isolated from poultry and cattle in Odisha, India. Infect Genet Evol. 2015;29: 82–90. doi: [10.1016/j.meegid.2014.11.003](https://doi.org/10.1016/j.meegid.2014.11.003)
10. Karuppasamy C, Ralte L, Malsawtluangi L, Chawang S. Prevalence of extended spectrum beta lactamase (esbl) producing pathogens in raw milk samples collected from Aizawl town, Mizoram. 2014 doi: 10.4172/2168-9547.1000201
11. Lalzampuia H, Dutta TK, Warjri I, Chandra R. Detection of extended-spectrum β-lactamases (blaCTX-M-1 and blaTEM. Vet World. 2014 Nov 1;7. doi: 10.14202/vetworld.2014.1026-31.
12. Lalzampuia H, Dutta TK, Warjri I, Chandra R. PCR-based detection of extended-spectrum β-lactamases (bla CTX-M-1 and bla TEM) in *Escherichia coli*, *Salmonella* spp. and *Klebsiella pneumoniae* isolated from pigs in North Eastern India (Mizoram). Indian J Microbiol. 2013 Sep 1;53(3):291-6. doi: [10.1007/s12088-013-0378-z](https://dx.doi.org/10.1007%2Fs12088-013-0378-z)
13. Mahanti A, Ghosh P, Samanta I, Joardar SN, Bandyopadhyay S, Bhattacharyya D, Banerjee J, Batabyal S, Sar TK, Dutta TK. Prevalence of CTX-M-Producing *Klebsiella* spp. in Broiler, Kuroiler, and Indigenous Poultry in West Bengal State, India. Microb Drug Res. 2018 Apr 1;24(3):299-306. doi: [10.1089/mdr.2016.0096](https://doi.org/10.1089/mdr.2016.0096)
14. Mandakini R, Dutta TK, Chingtham S, Roychoudhury P, Samanta I, Joardar SN, Pachauau AR, Chandra R. ESBL-producing Shiga-toxigenic *E. coli* (STEC) associated with piglet diarrhoea in India. Trop Anim Health Prod. 2015 Feb 1;47(2):377-81. doi: [10.1007/s11250-014-0731-1](https://doi.org/10.1007/s11250-014-0731-1)
15. Nirupama KR, OR VK, Pruthvishree BS, Sinha DK, Murugan MS, Krishnaswamy N, Singh BR. Molecular characterisation of blaOXA-48 carbapenemase-, extended-spectrum β-lactamase-and Shiga toxin-producing *Escherichia coli* isolated from farm piglets in India. J Global Antimicrob Res. 2018 Jun 1;13:201-5. doi.org/10.1016/j.jgar.2018.01.007
16. Raj JR, Vittal R, Shivakumaraswamy SK, Deekshit VK, Chakraborty A, Karunasagar I. Presence & mobility of antimicrobial resistance in Gram-negative bacteria from environmental samples in coastal Karnataka, India. Indian J Med Res. 2019 Feb 1;149(2):290. doi: 10.4103/ijmr.IJMR_2088_17
17. Rasheed MU, Thajuddin N, Ahamed P, Teklemariam Z, Jamil K. Antimicrobial drug resistance in strains of *Escherichia coli* isolated from food sources. Revista do Instituto de Medicina Tropical de São Paulo. 2014 Aug;56(4):341-6.
18. Sharif NM, Sreedevi B, Chaitanya RK, Srilatha C. Detection of extended spectrum beta-lactam (ESBL) resistance in *Pseudomonas* species of canine origin. Pharma Innov. 2017 Sep 1;6:89.
19. Tewari R, Mitra S, Ganaie F, Das S, Chakraborty A, Venugopal N, Shome R, Rahman H, Shome BR. Dissemination and characterization of extended spectrum β-lactamase, AmpC β-lactamase and metallo β-lactamase producing *Escherichia coli* from livestock and poultry in Northeastern India: A molecular surveillance approach. J Global Antimicrob Res. 2019 Jan 8; 17 (2019) 209–215. doi: doi.org/10.1016/j.jgar.2018.12.025
20. Tewari R, Mitra SD, Venugopal N, Das S, Ganaie F, Sen A, Shome R, Rahman H, Shome BR. Phenotypic and molecular characterization of extended spectrum β-lactamase, ampc β-lactamase and metallo β-lactamase producing *Klebsiella* spp. from farm animals in India. Indian J Anim Res. 2018 doi: 10.18805/ijar.B-3599
